# Crosslinked-Polymer Brushes with Switchable Capture and Release Capabilities

**DOI:** 10.3390/polym10090956

**Published:** 2018-08-29

**Authors:** Serkan Demirci

**Affiliations:** 1 Department of Chemistry, Amasya University, Ipekkoy, Amasya 05100, Turkey; srkndemirci@gmail.com or sdemirci@amasya.edu.tr; Tel.: +90-358-242-1614; 2 Department of Biotechnology, Amasya University, Ipekkoy, Amasya 05100, Turkey

**Keywords:** crosslinked-polymer brushes, brush gels, RAFT polymerization, cyclodextrin, responsive polymer

## Abstract

Crosslinked-polymer brushes give rise to new opportunities for functionalizing, protecting, and structuring both organic and inorganic materials. In this study, pH- and temperature-switchable crosslinked-polymer brushes were easily prepared by combining the in situ method with reversible addition–fragmentation chain transfer (RAFT) polymerization. Initially, the RAFT agent was immobilized on an amine-terminated silicon wafer surface and utilized in the surface-initiated RAFT polymerization of 2-*N*-morpholinoethyl methacrylate (MEMA) as a monomer, and β-cyclodextrin methacrylate (CDMA) was used as a crosslinker on the silicon substrate. Measurements of film thickness, water contact angle, surface morphology, and structural characteristics of the resulting surfaces confirmed the poly(2-*N*-morpholinoethyl methacrylate) (PMEMA) brush-gels. Reversible capture and release measurements of methylene blue as a model molecule were also performed by UV–vis analysis. The switchable properties of the PMEMA brush-gels were maintained over five cycles. The results indicate that these PMEMA brush-gels with reversible capture and release properties might have widespread potential applications, including improved diagnostic tools as well as bioseparation.

## 1. Introduction

Functional polymer films with stimuli-responsive behaviors have been widely investigated and are considered to be smart materials due to their potential application in a variety of areas including filtration/separation [1,2], biotechnology [3,4,5], and biomaterials [6,7]. Recent advances in polymer chemistry have allowed for the synthesis of a variety of functional polymer films such as the polymer brush, which is a monomolecular film in which each macromolecule is attached at one end to a solid surface. Polymer brushes with well-defined architectures, such as a homopolymer [8,9,10,11], block copolymer [12,13,14], and binary mixed polymer brushes [15,16,17], combined with different novel classes of materials, are being introduced continuously. Furthermore, hydrogels are another example of smart materials [18,19], which are versatile and tunable, and have gained considerable interest as biomedical and drug carrier devices [19].

As smart materials, polymer brushes and hydrogels have particular advantages and disadvantages. Crosslinked-polymer brushes, known as polymer brush-gels, have emerged as a new class in the field of materials [20]. Polymer brush-gels have some of the characteristics of both polymer brushes and gels. Compared with traditional polymer brush systems, polymer brush-gels have higher stability and functionality [21], and lower surface corrosion. Surface-grafted polymer-gels can be obtained either via post-functionalization reactions or during the polymerization process itself [21,22]. In addition, the crosslinking provides additional stability to the polymer brushes, improving the overall performance of polymer-coated surfaces. These brush-gels have potential for biomedical applications such as drug delivery, antifouling surface coating, enzyme immobilization, and tissue engineering [20].

Cyclodextrins (CDs) have been widely used in the preparation of hydrogels and nanogels as crosslinkers and host molecules. In covalently crosslinked systems, CDs are incorporated to improve the hydrogel characteristics, whereas in physically crosslinked systems, the inclusion of guest molecules in CD cavities is used as the driving force behind network formation. CDs are cyclic oligosaccharides with a toroid-shaped molecular structure most commonly consisting of 6-, 7-, or 8-glucose, which are α-, β-, and γ-CDs, respectively [23,24]. CDs are well-known host molecules in supramolecular chemistry. Due to their unique structure with hydrophobic interior cavities and hydrophilic exterior properties, CDs can form non-covalent host–guest inclusion complexes (IC) with a variety of molecules including drugs, antibacterials, food additives, etc. [23,24,25,26,27,28] The physical and chemical properties of these guest molecules are improved when complexed with CDs [28,29,30].

Numerous studies have described the preparation of CD-functionalized polymer brushes. For example, Chen et al. [31] demonstrated the preparation of poly(*N*-isopropylacrylamide-*co*-1-adamantan-1-ylmethyl acrylate)-modified surfaces via single-electron transfer living radical polymerization. Furthermore, β-CD-(mannose)_7_ was synthesized and complexed with adamantane molecules via host–guest interaction. The effect of adamantine and the CD-adamantane complex on the wettability and thermo-responsive properties of surfaces was investigated. The use of cyclodextrin/azobenzene/polymer for the reversible immobilization of biomolecules was reported by Cheng et al. [32] They showed the light-triggered switching of reversible and alterable biofunctionality on a silicon interface via host–guest interaction. In 2011, Wang and coworkers reviewed a new strategy for the preparation of a dually functionalized poly(dimethylsiloxane) surface using surface-initiated atom transfer radical polymerization and click chemistry [33]. Initially, poly(ethylene glycol) brushes were synthesized in which the CD-functionalized surface was then prepared via a click reaction. These prepared surfaces were used in a sandwich fluoroimmunoassay of cardiac markers myoglobin and fatty acid-binding protein.

Despite the stability of crosslinked-polymer brushes and the unique properties of cyclodextrins, no studies have dealt with polymer brush-gels developed by crosslinking polymer brushes with cyclodextrins. Instead of the abovementioned CD-functionalized polymer brushes, a new strategy for the preparation of stable, multifunctional, and tunable crosslinked-polymer brushes is reported in this paper. pH- and temperature-responsive poly(2-*N*-morpholinoethyl methacrylate) (PMEMA) brush-gels were synthesized with different crosslinker ratios, combining the in situ method with RAFT polymerization. The brush-gels were characterized by FTIR, XPS, AFM, ellipsometry, and static water contact angle measurements. PMEMA brush-gels were shown to be well-rounded and chemically stable polymeric films, with switchable capture and release properties at a nearly constant chemical composition. The results showed that surface functionalization with this class of brush layer could represent a promising coating approach to independently tailor the chemical and mechanical properties of a variety of materials.

## 2. Materials and Methods

### 2.1. Materials

2-*N*-morpholinoethyl methacrylate (MEMA, 95%, Aldrich, St. Louis, MO, USA), styrene (99.9%, Aldrich), 4-cyano-4-(phenylcarbonothioylthio)pentanoic acid (CPDB, >97%, Aldrich), 4-cyano-4-(phenylcarbonothioylthio)pentanoic acid *N*-succinimidyl ester (CPSE, Aldrich), 4,4′-azobis(4-cyanovaleric acid) (ACVA, ≥98.0%, Aldrich), hydrogen peroxide (30 wt % in water, Sigma-Aldrich), sodium hydroxide (≥98%, Sigma-Aldrich), magnesium sulfate (≥99.5%, Sigma-Aldrich), ammonium hydroxide (28.0%–30.0%, Sigma-Aldrich), trifluoroacetic acid (TFA, 99%, Sigma-Aldrich), ammonium hydroxide (28.0%–30.0%, Sigma-Aldrich), di-tert-butyl decarbonate ( ≥98%, Sigma-Aldrich) and acetone (≥99.8%, Sigma-Aldrich) were used as received without further purification. Chloroform (≥99.5%, Sigma-Aldrich), acetonitrile (MeCN, 99.8%, Sigma-Aldrich), dichloromethane (DCM, ≥99.8%, Sigma-Aldrich), ethyl acetate (EtOAc, ≥99.7%, Sigma-Aldrich), *N*,*N*-dimethylformamide (DMF, 99.8%, Sigma-Aldrich), diethyl ether (Et_2_O, ≥99.0%, Sigma-Aldrich), tetrahydrofuran (THF, ≥99.9%, Sigma-Aldrich) and methacryloyl chloride (97%, Aldrich) were purified according to published protocols. Silicon (100) wafers (single side polished, *N*-type) were purchased from Aldrich. β-Cyclodextrin (CD) was kindly donated by Roquette (Lestrem, France). Deionized water (18.2 MΩ⋅cm) was obtained from a Milli-Q water purification system (Millipore, Bedford, MA, USA).

### 2.2. Synthesis of Cyclodextrin Methacrylate (CDMA)

The preparation of CDMA was as previously described in the literature, with minor modifications [34]. Briefly, under a nitrogen atmosphere, CD (2 g, 1.76 mmol) was dissolved in 15 mL DMF, and triethylamine (0.45 g, 4.40 mmol) was added dropwise to the solution at 0 °C. Methacryloyl chloride (0.55 g, 5.28 mmol) was subsequently added dropwise to the reaction mixture. The mixture was stirred at room temperature for 16 h. A precipitate was filtrated out, and the filtrate was poured into cold acetone (250 mL). A white precipitate of the product was obtained after filtration. Yield: 65.3%. ^1^H NMR (600 MHz, DMSO-*d_6_*, δ, ppm): 6.05 (m, 2H), 5.79 (m, 2H), 5.80–5.67 (b, 14H), 4.90–4.81 (b, 7H), 4.58–4.40 (b, 5H), 3.80–3.49 (b, 42H), 1.84 (m, 6H). FTIR (ATR-FTIR) *υ* (cm^−1^): 3500–3200 (b, O–H), 1720 (s, C=O), 1155 (s, O–C–O), 1080 (C–O), 1030 (C–O–C).

### 2.3. Surface-Initiated Polymerization

The preparation of the RAFT-agent-immobilized silicon wafers (Si–RAFT) was performed according to a previously published protocol [35,36]. The silicon wafers were initially cleaned in a “piranha” solution, which is a 3:1 mixture of concentrated H_2_SO_4_ and H_2_O_2_ (30%) heated to 90 °C for 30 min (CAUTION: “piranha” solution reacts violently with organic materials and must be handled with extreme care), followed by copious rinsing with deionized water. The cleaned silicon wafers were etched for 1 min in a 2% hydrofluoric acid solution, quickly rinsed in degassed deionized water, and dried in a stream of nitrogen. *t*-Butyloxycarbonyl (*t*-BOC)-protected allylamine (20 μL), which was prepared as per the standard method [37], was introduced onto the freshly prepared Si–H wafers. The prepared sample was sandwiched between two quartz plates, and a uniform thin liquid film of *t*-BOC-protected allylamine formed on the Si–H wafers. The silicon wafer was placed in a N_2_ purged steel reaction chamber covered with a quartz window and irradiated with UV light (λ = 254 nm) for 2 h. After irradiation, the modified silicon wafers were ultra-sonically washed with 25% TFA in methylene chloride, followed by a 1-min rinse in 10% NH_4_OH to remove the *t*-BOC protecting group and dried in a stream of nitrogen. The Si–NH_2_ silicon wafers were placed into a solution of CPSE (4.70 g, 12.5 mmol) in 50 mL of freshly distilled DCM. The reaction mixture was left to react at ambient temperature for 60 h. The silicon wafers were recovered from the reaction mixture, repeatedly washed with DCM and acetone, and dried under a stream of nitrogen.

The RAFT-agent-immobilized silicon wafers were placed in a glass reactor and degassed under vacuum for 30 min. MEMA monomer (4.18 g, 21.0 mmol) and CDMA crosslinker (1%, 2%, and 4% monomer) were added to a dry Schlenk tube along with CPDB (27.9 mg, 0.1 mmol), ACVA (14.0 mg, 0.05 mmol), and DMF (10 mL). The mixture was degassed in three freeze-pump-thaw cycles. The solution was transferred to a glass reactor just before heating the reactor to 70 °C for 24 h to allow polymerization. After quenching the reaction in an ice bath, the modified silicon wafers were washed with acetone and dried under a stream of nitrogen.

### 2.4. Measurement of Methylene Blue Capture and Release

**Scheme 1:**
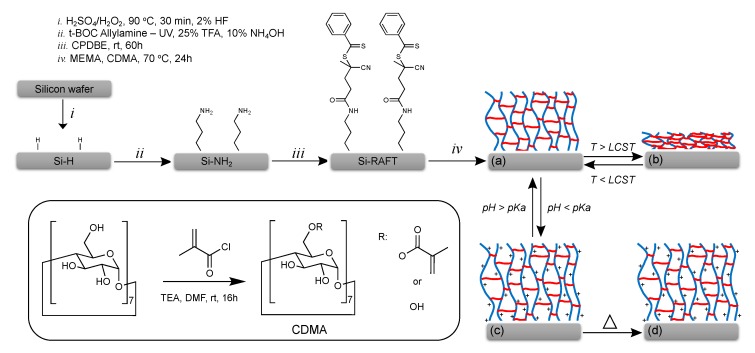
Synthesis of β-cyclodextrin methacrylate (CDMA) and schematic procedure for fabricating poly(2-*N*-morpholinoethyl methacrylate) (PMEMA) brush-gels: (**a**) PMEMA brush-gels: capture and release; (**b**) collapsed-PMEMA brush-gels: no capture and no release; (**c**) cationic PMEMA brush-gels: release; (**d**) cationic PMEMA brush-gels: fast release.

Methylene blue was used as a target molecule for the determination of the switchable capabilities of PMEMA brush-gels. The silicon wafers coated with PMEMA brush-gels (Scheme 1a–d), which were prepared with different crosslinker ratios, were immersed in an aqueous solution (25 μm) of methylene blue at room temperature and 40 °C. The depletion of methylene blue from the solution was determined by UV–vis spectrophotometry at 663 nm. The PMEMA brush-gels were loaded with methylene blue under maximum adsorption conditions. Subsequently, methylene blue release from the PMEMA brush-gels was followed under diverse environmental conditions with UV–vis spectrophotometry at 663 nm.

In order to test the reusability of the PMEMA brush-gels for methylene blue capture and release, five cycles of capture/release were carried out. After each capture/release cycle, capture/release efficiency was determined by UV–vis spectrophotometry at 663 nm.

### 2.5. Characterization

Proton nuclear magnetic resonance (^1^H NMR) was performed on a Bruker Avance III (Bruker BioSpin GmbH, Rheinstetten, Germany) at 600 MHz. Samples were measured at room temperature in DMSO-*d_6_*. Fourier transform infrared (FTIR) spectra were recorded on a Nicolet 6700 instrument (Thermo Scientific, Waltham, MA, USA) with a Smart Orbit attenuated total reflection attachment. The spectra were taken at a resolution 4 cm^−1^ after a 128-scan accumulation for an acceptable signal/noise ratio. Visible absorption spectra were obtained using a Perkin-Elmer Lambda 35 UV–vis spectrophotometer (Perkin-Elmer, Norwalk, CT, USA). The absorption spectrum of the methylene blue solution was recorded using deionized (DI) water as the solvent. The chemical composition information of the samples was obtained by X-ray photoelectron spectroscopy (XPS); the measurement was carried out on a Thermo Scientific K-Alpha X-ray photoelectron spectrometer (Thermo Scientific, Waltham, MA, USA) using a monochromatic Al K-α X-ray source (hν = 1486.6 eV). Charging neutralizing equipment was used to compensate sample charging, and the binding scale was referenced to the aliphatic component of the C 1s spectrum at 285.0 eV. The water contact angle measurements were conducted at room temperature using a drop shape analyzer (model DSA100, Krüss, Hamburg, Germany) equipped with a microliter syringe. Deionized water (5.0 μL) was used as the wetting liquid. The morphology of the silicon wafers was recorded with an atomic force microscope (AFM, model XE-70, Park Systems, South Korea). A triangular-shaped Si_3_N_4_ cantilever with integrated tips (Olympus, Tokyo, Japan) was used to acquire the images in the non-contact mode. The normal spring constant of the cantilever was 0.02 N/m. The force between the tip and the sample was 0.87 nN. The ellipsometric measurements were performed under ambient conditions using a monochromatic ellipsometer (model EL X-02C, DRE Dr. Riss Ellipsometerbau GmbH, Ratzeburg, Germany) equipped with a He-Ne laser (λ = 632.8 nm) at a constant incident angle of 75°. The average dry thickness of the polymer brushes on the silicon substrate was determined by fitting the data with a three-layer model (native silicon (refractive index, *n* = 3.86) + silicon oxide layer (*n* = 1.46) + organic layer (*n* = 1.47)) [38,39].

## 3. Results and Discussions

### 3.1. Surface Modification and Characterization

As illustrated in Scheme 1, silicon wafers were cleaned and modified with allylamine by UV irradiation. Subsequently, the RAFT agent was immobilized on Si–NH_2_ surfaces through an amide reaction. Crosslinked-polymer brushes were prepared combining the in situ method with RAFT polymerization. Finally, the capture and release of methylene blue were performed under different environmental conditions. For the sake of clarity, brush-gels obtained with different crosslinker ratios are named PMEMA–CDMA–1, PMEMA–CDMA–2, and PMEMA–CDMA–4, indicating brush-gels synthesized with 1, 2, and 4 mol % CDMA, respectively.

**Figure 1:**
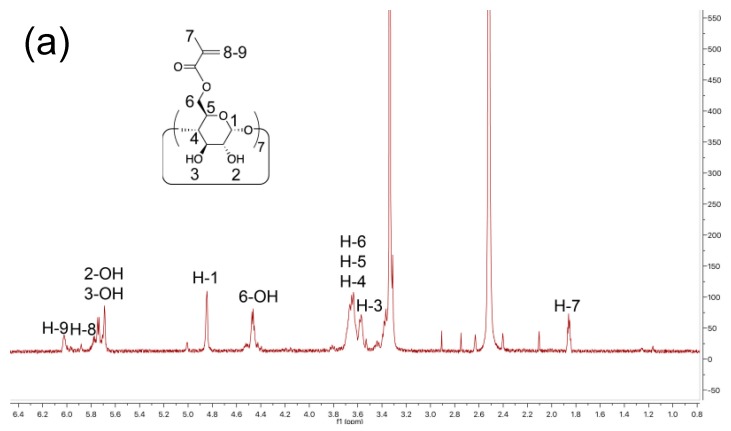

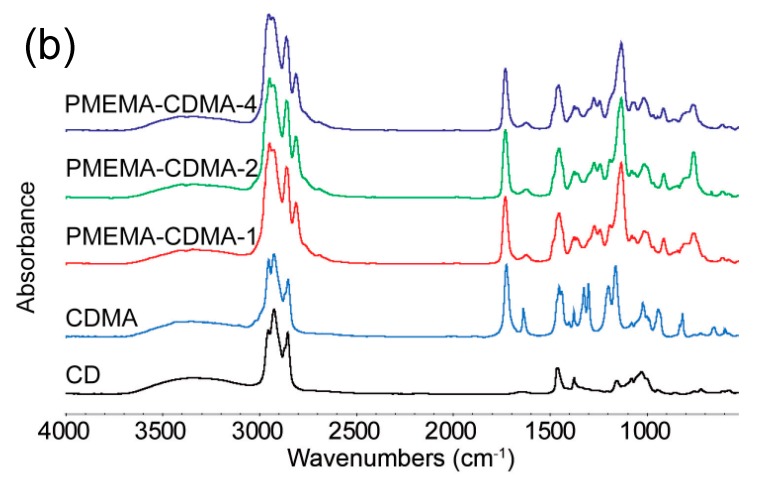
^1^H NMR spectrum of CDMA (**a**); FTIR spectra of β-cyclodextrin (CD), CDMA, and PMEMA–CDMA brush-gels (**b**).

In the ^1^H NMR spectrum of the CDMA (Figure 1a), the characteristic protons on the cyclodextrin unit appeared at approximately 5.80, 4.85, 4.55, and 3.55 ppm. The vinyl and methyl protons were observed at 6.05, 5.79, and 1.84 ppm. The formation of PMEMA brush-gels was confirmed using FTIR (Figure 1b). The characteristic bands of PMEMA were absorbed at 1730 and 1133 cm^−1^ due to the C=O and C–O–C stretching. As seen, the characteristic absorption bands of CD for the three given samples appeared at around 1027, 1079, and 1155 cm^−1^, respectively, corresponding to the coupled C–C/C–O stretching vibrations and asymmetric stretching vibration of the C–O–C glycosidic bridge. The C–O–C vibration at 1020 cm^−1^ is a unique band and characterizes the presence of CDMA in the PMEMA brush-gels. The conversion of the monomer was determined by FTIR. After 24 h, the monomer conversions were almost 82%, 84%, and 81% for PMEMA–CDMA–1, PMEMA–CDMA–2, and PMEMA–CDMA–4, respectively.

The surfaces of Si–NH_2_, Si–RAFT, and crosslinked-polymer brushes were characterized using XPS wide and high-resolution scans to verify the functionalization of these samples. Table 1 summarizes the compositional percentages of modified silicon wafer surfaces which were obtained using a wide energy survey scan. The XPS analysis of the amine-terminated layer verified the presence of C 1s and N 1s. The immobilization of the RAFT agent onto the Si–NH_2_ layer was confirmed by the appearance of the S 2p peak (Table 1). The XPS results of Si–NH_2_ and Si–RAFT were compatible with the literature values [35,36]. It was observed that O 1s, N 1s, C 1s, and S 2p are four intensive elements in the main composition of the PMEMA brush-gels. The ratio of O 1s increased with the crosslinker ratio, whereas those of C 1s and N 1s decreased due to the contribution of CDMA. A high-resolution C 1s scan was performed to obtain more detailed information about the chemical state of the brush-gel surface. The C 1s spectra of the PMEMA brush-gels could be curve-fitted to five peak components. The corresponding positions of peak binding energies are also listed in Table 1.

**Figure 2:**
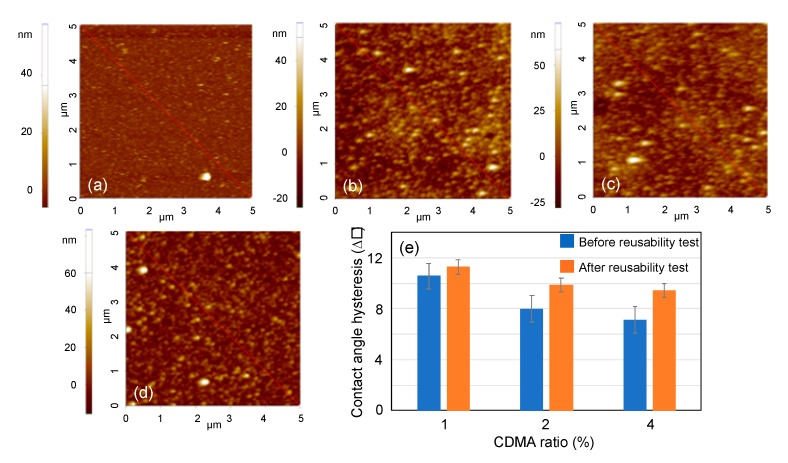
AFM images of Si–RAFT (**a**); PMEMA–CDMA–1 (**b**); PMEMA–CDMA–2 (**c**); PMEMA–CDMA–4 (**d**); Water contact angle hysteresis of PMEMA brush-gels (**e**).

The surface morphology of the PMEMA brush-gels was determined by AFM as depicted in Figure 2. The comparison with the RAFT-agent-modified surface (root mean square (RMS) roughness = 1.314 nm) evidenced significant changes in topography and surface roughness resulting from the PMEMA brush-gel formation. For the PMEMA brush-gels, the film morphologies appeared as needle-like structures heterogeneously distributed over the entire substrate area, and the RMS roughnesses were 6.102, 6.048, and 5.170 nm for PMEMA–CDMA–1, PMEMA–CDMA–2, and PMEMA–CDMA–4, respectively. The change in the film morphology with the crosslinker ratio was associated with a decrease in the RMS roughness. AFM results were confirmed with contact angle hysteresis (advancing angle (θ_a_) – receding angle (θ_r_) = Δθ) results (Figure 2e). It can be seen that the hysteresis decreases with increasing crosslinker ratio. These results point out that the roughness of the PMEMA brush-gels decreases with increasing CDMA ratio. The water contact angles of the brush-gels were found to be 31° ± 3°, 34° ± 2°, and 38° ± 3°, for PMEMA–CDMA–1, PMEMA–CDMA–2, and PMEMA–CDMA–4, respectively, meaning that the surfaces are hydrophilic. The ellipsometric thicknesses of the PMEMA brush-gels were measured to be 34 ± 5, 39 ± 6, and 43 ± 3 nm for PMEMA–CDMA–1, PMEMA–CDMA–2, and PMEMA–CDMA–4, respectively. All the results clearly show that PMEMA brush-gels were successfully prepared.

### 3.2. Reversible Capture and Release

Methylene blue, a synthetic basic dye, was chosen as a model molecule for the reversible capture and release ability of PMEMA brush-gels. As it is known, CDs are capable of encapsulating organic molecules and there are many studies reporting complexation between CDs and dye molecules [34]. PMEMA has pH- (pKa = 4.9) [40] and temperature-responsive (lower critical solution temperature, LCST = 37 °C) properties [41]. The influence of pH and temperature on methylene blue capture and release was determined by performing the adsorption-desorption experiments below and above the pKa volume of PMEMA at room temperature and 40 °C.

**Figure 3:**
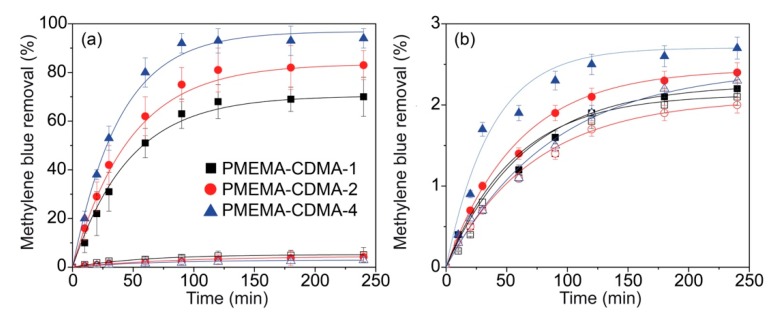
Variation of methylene blue removal with contact time for the adsorption of methylene blue onto PMEMA brush gels under pH > pKa (**a**) and pH < pKa (**b**) conditions at room temperature (filled symbols) and 40 °C (open symbols).

As shown in Figure 3, the adsorption of methylene blue was unfavorable at pH < pKa and increased with increased pH. This is because methylene blue is a basic dye and PMEMA is cationic below the pKa value. The PMEMA chains lost their thermo-responsive property under acidic conditions. As a result, there were no significant differences in the results between the two temperatures. However, above the pKa value, the PMEMA brush-gels maintained their abilities. With increased pH, the PMEMA chains became neutral and allowed the dye adsorption by CD molecules at room temperature. Moreover, the adsorption increased with increasing crosslinker ratio. The PMEMA brush-gels shrunk when the temperature increased from room temperature to 40 °C, due to the tendency of PMEMA to aggregate above its lower critical solution temperature (37 °C). Collapsed PMEMA blocked the CD cavities and did not allow dye adsorption. PMEMA brush-gels showed high adsorption capacities at room temperature and higher pH conditions (> pKa). The pH- and temperature-responsive behavior of brush-gels is illustrated in Scheme 1.

**Figure 4:**
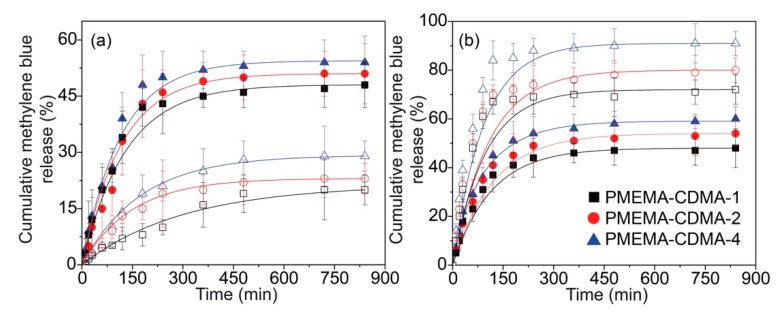
Release profiles of methylene blue from PMEMA brush-gels under pH > pKa (**a**) and pH < pKa (**b**) conditions at room temperature (filled symbols) and 40 °C (open symbols).

Loading efficiency is an important parameter to measure the performance of the PMEMA brush-gels, and was obtained from the absorbance intensity of the methylene blue solution. The PMEMA brush-gels were loaded with methylene blue under maximum adsorption conditions (pH > pKa and room temperature). The loading efficiencies of the PMEMA brush-gels loaded with 5% (*w*/*w*, with respect to polymer) methylene blue were determined to be 70% ± 8%, 83% ± 6%, and 95% ± 7% for PMEMA–CDMA–1, PMEMA–CDMA–2, and PMEMA–CDMA–4, respectively. Swelling of the resulting brush-gels was dependent on both temperature and pH. The dye release from the gels was investigated at pH 4.01 and 7.4 at 24 and 40 °C. The methylene blue release was characterized by UV–vis spectra. Figure 4 shows the cumulative methylene blue release (%) from the PMEMA brush-gels encapsulating methylene blue under different environmental conditions. Two stages of release can be distinguished in the release profiles; after a quick initial release that continued for 120 min, the following time interval showed a sustained release of methylene blue from the PMEMA brush-gels, except for PMEMA brush-gels at 40 °C.

The absorption intensity gradually increased, which indicates the increasing methylene blue release from PMEMA brush-gels with decreasing pH. As mentioned above, the PMEMA chains became cationic at low pH values, and methylene blue, which is a positively charged model molecule, was pushed from the PMEMA brush-gels at room temperature. The release of methylene blue from the cationic PMEMA brush-gels at 40 °C was about 1.8 times higher than that from cationic PMEMA brush-gels at room temperature, and about twice as high as that from PMEMA brush-gels at room temperature. The faster release of methylene blue from the cationic PMEMA brush-gels compared to PMEMA brush-gels was attributed to the electrostatic interaction between methylene blue and the PMEMA chains. Methylene blue is positively charged, which leads to a repulsion of the PMEMA chains. In addition, the increasing temperature caused the dissociation of methylene blue/CD inclusion complexes and increased the dye release.

**Figure 5:**
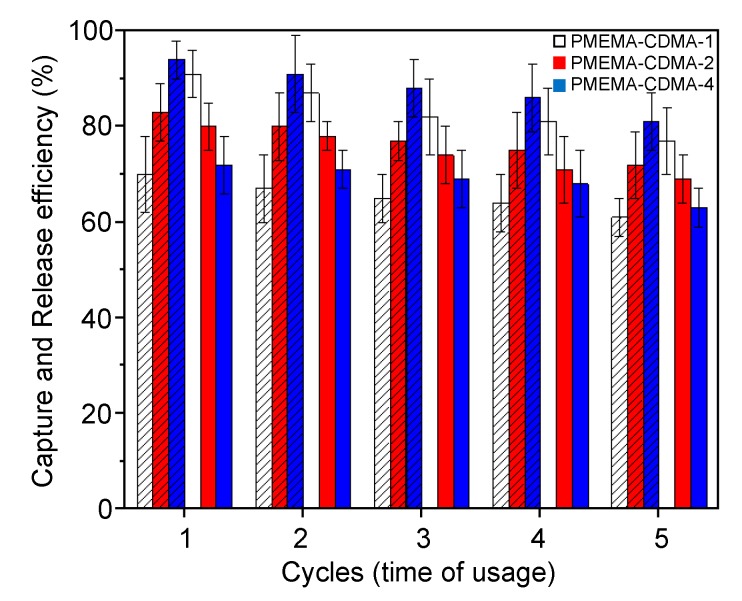
Capture (patterned) and release efficiency of PMEMA brush-gels with methylene blue in repeated cycles.

The reusability of the PMEMA brush-gels was investigated by measuring the capture and release capacity for the target dye molecule in a cyclic manner (Figure 5). The capture and release procedures were repeated five times to verify the reusability of the PMEMA brush-gels. As shown in Figure 5, the PMEMA brush-gels can still be reused. However, the adsorption and release capacities of the PMEMA brush-gels decreased with increasing crosslinker ratio. For the PMEMA brush-gels incorporating 4% crosslinker, a decrease (approximately 2%) in the capture and release capacities was seen during each cycle, and brush-gels retained methylene blue with an uptake capacity of 14% after five cycles.

After the reusability test, the contact angle hysteresis of the PMEMA brush-gels showed a slight increase (Figure 2e). Similar to previous results, the hysteresis decreased with the CDMA ratio. These results point out that there are no significant changes in surface morphology after five capture and release cycles; thus, these PMEMA brush gels can be repeatedly used without sacrificing the surface morphology and functionality.

## 4. Conclusions

In conclusion, PMEMA brush-gels with switchable properties were successfully prepared combining the in situ method with RAFT polymerization. Techniques such as FTIR, XPS, ellipsometry, and AFM confirmed the presence of the PMEMA brush-gels. The pH- and temperature-responsive behavior of the PMEMA brush-gels was also investigated by analyzing the host-guest interaction between methylene blue and CD under different environmental conditions. The release of methylene blue from cationic PMEMA brush-gels was about twice as high as that from PMEMA brush-gels. The PMEMA chains lost their thermo-responsive property under acidic conditions. However, under neutral and basic conditions they maintained their abilities. The results showed that behavior and complexation could be controlled and switched. The crosslinked-polymer brushes may provide additional stability and improve the overall performance of polymer-coated surfaces for further applications. Additionally, these newly developed brush-gels may be potentially useful for bioseparation, biosensors, and biomedical engineering via supramolecular interaction.

## Figures and Tables

**Table 1:** Atomic concentration (Conc.) and binding energies obtained from high-resolution X-ray photoelectron spectroscopy (XPS) for PMEMA brush-gels ^1^.

Sample		O 1s	N 1s	C 1s					S 2p		Si 2p
				O–C=O	O–C–O	C–O	C–N	C–(C–H)	S–C	S=C	
**Si–NH_2_**	Energy (eV) Conc. (%)	- -	400.0 19.7	- 64.1	- -	- -	285.7	285.0	- -	- -	99.1 16.2
**Si–RAFT**	Energy (eV) Conc. (%)	532.6 4.3	400.1 12.5	289.2 66.7	-	-	285.7	285.0	163.8 7.3	162.4	99.1 9.2
**PMEMA–CDMA–1**	Energy (eV) Conc. (%)	532.1 21.9	400.1 7.1	289.2 69.7	287.6	286.5	285.7	285.0	163.9 1.3	162.3	- -
**PMEMA–CDMA–2**	Energy (eV) Conc. (%)	532.2 27.7	400.1 5.8	289.3 65.6	287.8	286.7	285.3	285.0	163.7 0.9	162.4	- -
**PMEMA–CDMA–4**	Energy (eV) Conc. (%)	532.1 30.7	400.2 4.4	289.1 64.1	287.9	286.6	285.5	285.0	163.9 0.8	162.2	- -

^1^ Binding energies were calibrated to aliphatic carbon at 285.0 eV.
